# Disruption of *pknG* enhances production of gamma-aminobutyric acid by *Corynebacterium glutamicum* expressing glutamate decarboxylase

**DOI:** 10.1186/s13568-014-0020-4

**Published:** 2014-04-01

**Authors:** Naoko Okai, Chihiro Takahashi, Kazuki Hatada, Chiaki Ogino, Akihiko Kondo

**Affiliations:** 1 Organization of Advanced Science and Technology, Kobe University, 1-1 Rokkodaicho, Nada-ku, Kobe 657-8501, Japan; 2 Department of Chemical Science and Engineering, Graduate School of Engineering, Kobe University, 1-1 Rokkodaicho, Nada-ku, Kobe 657-8501, Japan

**Keywords:** Corynebacterium glutamicum, Gamma-aminobutyric acid, Glutamate decarboxylase, 2-oxoglutarate dehydrogenase, Protein kinase G

## Abstract

Gamma-aminobutyric acid (GABA), a building block of the biodegradable plastic polyamide 4, is synthesized from glucose by *Corynebacterium glutamicum* that expresses *Escherichia coli* glutamate decarboxylase (GAD) B encoded by *gadB*. This strain was engineered to produce GABA more efficiently from biomass-derived sugars. To enhance GABA production further by increasing the intracellular concentration of its precursor glutamate, we focused on engineering *pknG* (encoding serine/threonine protein kinase G), which controls the activity of 2-oxoglutarate dehydrogenase (Odh) in the tricarboxylic acid cycle branch point leading to glutamate synthesis. We succeeded in expressing GadB in a *C. glutamicum* strain harboring a deletion of *pknG. C. glutamicum* strains GAD and GAD ∆*pknG* were cultured in GP2 medium containing 100 g L^−1^ glucose and 0.1 mM pyridoxal 5′-phosphate. Strain GAD∆*pknG* produced 31.1 ± 0.41 g L^−1^ (0.259 g L^−1^ h^−1^) of GABA in 120 hours, representing a 2.29-fold higher level compared with GAD. The production yield of GABA from glucose by GAD∆*pknG* reached 0.893 mol mol^−1^.

## Introduction

Diverse microorganisms, animals, and plants synthesize the amino acid gamma-aminobutyric acid (GABA), which does not naturally occur in proteins. GABA functions as a neurotransmitter in humans, lowers blood pressure (Hayakawa et al. [2004]), and is a component of pharmaceuticals and foods (Li and Cao [2010]). The bioplastic polyamide 4 (PA4) is a linear polymer of GABA, which is chemically synthesized from the GABA lactam 2-pyrrolidone (Kawasaki et al. [2005]). PA4 has excellent physical properties based on its high melting point of 260°C and its degradability by microbes in soil (Hashimoto et al. [1994]) and activated sludge (Yamano et al. [2008]). The synthesis of GABA from abundantly available biomass by recombinant microorganisms will make it possible to produce new bioplastics at low cost.

Glutamate decarboxylase (GAD; EC 4.1.1.15) catalyzes the conversion of L-glutamate to GABA through alpha-decarboxylation (Fonda [1985]). Genes (*gad*) encoding GAD are present in *Escherichia coli* (DeBiase et al. [1996]), *Lactobacillus brevis* (Oda et al. [2008]), *Lactobacillus paracasei* (Shima et al. [2008]), and several other species of *Lactobacillus* as well as *Enterobacteria.* Lactic acid bacteria produce GABA when glutamate is added to the fermentation medium. Although the quantities of GABA produced by this method are sufficient for producing foods, it is not cost-effective for producing chemicals.

Therefore, we developed a robust system for producing GABA from saccharides by expressing *E. coli* GAD in *Corynebacterium glutamicum* (Takahashi et al. [2012]). The biotin auxotroph *C. glutamicum* is a nonpathogenic, nonsporulating, nonmotile, Gram-positive soil bacterium that belongs to the order *Actinomycetales*, which includes *Corynebacteria*, *Nocardia*, *Rhodococci*, and other related microorganisms (George [2001]). *C. glutamicum* is an important industrial microorganism, because it produces high levels of glutamate and other amino acids, which are widely used in pharmaceuticals, animal feed, and food supplements (Leuchtenberger et al. [2005], Hermann [2003]). To efficiently produce GABA, the *gadB* gene from *E. coli* W3110 was overexpressed in a glutamate-producing *C. glutamicum* strain (ATCC 13032). After optimization, this strain produced 12.37 g L^−1^ of GABA from glucose in the presence of pyridoxal 5′-phosphate in the absence of added glutamate (Takahashi et al. [2012]).

In the present study, we further enhanced GABA synthesis using recombinant *C. glutamicum* strains expressing GAD to optimize the intracellular level of glutamate. To increase the flux of 2-oxoglutarate to glutamate, we attempted to disrupt *pknG*, which affects the activity of 2-oxoglutarate dehydrogenase (Odh). The reduction of the 2-oxoglutarate dehydrogenase complex (ODHC) is an important factor for glutamate synthesis by *C. glutamicum* (Kimura [2002]). ODHC participates in the tricarboxylic acid (TCA) cycle and catalyzes the conversion of 2-oxoglutarate to succinyl-CoA. The ODHC comprises four subunits, OdhI, OdhA, AceF, and Lpd. The activity of *C. glutamicum* ODHC is controlled by a regulatory mechanism that involves OdhI and serine/threonine protein kinase G (PknG, EC 2.7.11.1) (Schultz et al. [2009]). PknG catalyzes the phosphorylation of OdhI, a 15 kDa subunit of ODHC. Unphosphorylated OdhI binds the EI subunit (OdhA) of ODHC and inhibits its activity. Inhibition of ODHC activity is reversed by phosphorylation of OdhI at threonine residue 14 by PknG (Niebisch et al. [2006]). The *pknG*-deficient mutant produces glutamate at a higher rate compared with the parental *C. glutamicum* strain, suggesting that the mutations influence ODHC activities (Schultz et al. [2007], Boulahya et al. [2010]).

In the present study, a *pknG*-deficient *C. glutamicum* strain expressing GAD was generated to increase the flux of 2-oxoglutarate towards glutamate for more efficient biosynthesis of GABA. Using this strain, we were able to produce significantly higher levels of GABA from glucose.

## Materials and methods

### Bacterial strains and media

The bacterial strains and plasmids used in this study are listed in Table 1. *E. coli* strains were grown in Luria-Bertani medium (10 g L^−1^ tryptone, 5 g L^−1^ yeast extract, and 5 g L^−1^ sodium chloride) containing 50 μg mL^−1^ kanamycin at 37°C. *C. glutamicum* ATCC 13032 and all recombinant strains were grown in brain–heart infusion (BHI) medium (Becton, Dickinson and Co., Franklin Lakes, NJ, USA). BHI medium supplemented with 25 μg mL^−1^ kanamycin and 1.5% agar was used to select *C. glutamicum* transformants. The transformants were first cultivated at 30°C for 24 hours in a test tube containing 5 mL BHI medium with 25 μg mL^−1^ kanamycin and then inoculated into 20 mL GP2 medium (Takahashi et al. [2012]) containing 25 μg mL^−1^ kanamycin in a 200-mL flask for fermentation.

**Table 1:** Bacterial strains and plasmids

**Bacterial strains or plasmids**	**Relevant characteristics**	**Reference or source**
*E. coli*		
SCS110	*rpsL* (Str^r^) *thr leu endA thi-l lacY galK galT ara tonA tsx dam dcm*	Stratagene
	*supE44Δ* (*lac-proAB*) [*F’traD36 proAB lacl*^*q*^*ZΔM15*]	
*C. glutamicum*		
ATCC 13032	Wild-type *C. glutamicum*, biotin-auxotrophic, L-glutamate producing strain	ATCC
W	Wild-type *C. glutamicum* derivative harboring pCH	Takahashi et al. [2012]
GAD	Wild-type *C. glutamicum* derivative harboring pCH-gadB	Takahashi et al. [2012]
Δ*pknG*	Wild-type *C. glutamicum* derivative with deletion in *pknG*	This study
GADΔ*pknG*	Wild-type *C. glutamicum* derivative with deletion in *pknG*, harboring pCH-gadB	This study
Plasmids		
pCH	*E. coli*-*C. glutamicum* shuttle vector with HCE promoter, Km^r^	Tateno et al. [2007]
pCH-gadB	pCH containing *gadB* from *E.coli* W3110, Km^r^	Takahashi et al. [2012]
pTM44	pHSG298 with *B. subtilis sacB* and *C. glutamicum hom*, Km^r^	Mimitsuka et al. [2007]
pTM44-ΔpknG	pTM44 with 1,601-bp *Sph*I-*Bam*HI fragment with deletions of *pknG*, Km^r^	This study

^r^ antibiotic resistance.

### Molecular genetic techniques

*E. coli* SCS110 was used to avoid DNA methylation, and polymerase chain reaction (PCR) was conducted using KOD-Plus2 DNA polymerase (Toyobo, Osaka, Japan). Plasmid DNA was purified using a LaboPass™ Plasmid Mini Purification Kit (Cosmo Bio Co., Ltd., Tokyo, Japan).

### Construction of plasmids for disrupting *pknG*

Genomic DNA was purified using the Wizard Genomic DNA Purification Kit (Promega, Madison, WI, USA) from *C. glutamicum* ATCC 13032 grown in BHI medium. The oligonucleotide primers used in this study are listed in Table 2. To inactivate *pknG* (KEGG Entry NCgl 2655, Gene Name Cgl 2751, 2469 nt), an 801-base pair (bp) upstream region of *pknG* (pknG-up) was amplified using PCR with the primer pair pknG-up-In-F and pknG-up-In-R-801 and strain ATCC 13032 genomic DNA as template. An 800-bp downstream region of *pknG* (pknG-down) was amplified using PCR with the primer pair pknG-up-In-F-1669 and pknG-down-In-R. The two fragments, pknG-up and pknG-down, were fused using overwrap PCR using the primer pair pknG-In-F2 and pknG-In-R2, yielding a 1,601-bp fragment of *pknG*. The amplified DNA fragment was purified from a 1.0% agarose gel using the Wizard SV Gel and PCR Clean-Up systems (Promega). The plasmid pTM44 (Mimitsuka et al. [2007]), which contains *sacB* from *B. subtilis*, was used as a suicide vector for markerless gene disruption and was digested with *Sph*I and *Bam*HI (New England BioLabs Inc., MA, USA) to remove a 1,344-bp *Sph*I-*Bam*HI fragment. The 1.60-kbp Δ*pknG* fragment was inserted into *Sph*I*-Bam*HI*-*digested pTM44 using an In Fusion HD Cloning Kit (Clontech Laboratories Inc., Mountain View, CA, USA), yielding pTM44-ΔpknG. The DNA sequences of the constructs were determined using an ABI PRISM 3100 Genetic Analyzer (Life Technologies, Carlsbad, CA, USA).

**Table 2:** Oligonucleotide primers

**Primer name**	**Sequence (5′- 3′)**	**Restriction enzyme**
pknG-up-In-F	GCCAAGCTTgcatgcATGAAGGATAATGAAGATTTCGATCCAGATTCACCAGC	*Sph*I
pknG-up-In-R-801	*ACCATTTGTGTCGCC*GGCTTTGCAGCGGTCTTTCAGGGA	
pknG-down-In-F-1669	GCCAAGCTTgcatgc*GGCGACACAAATGGT*TCTCCG	*Sph*I
pknG-down-In-R	AAAAGGATCggatccCTAGAACCAACTCAGTGGCCGCA	*Bam*HI
pknG-In-F2	CCAGTGCCAAGCTTgcatgcATGAAGGATAATGAAGATTTCGATCCAGATTCACCAGC	*Sph*I
pknG-In-R2	AAAAAGGATCggatccCTAGAACCAACTCAGTGGCCGCA	*Bam*HI
SacI-gadB-F	GGCgagctcATGTTTAAAGCTGTTCTGTTGGGCAA	*Sac*I
XhoI-gadBF-R	CCGctcgagTTACTTGTCATCGTCATCCTTGTAGTCAGGTCGGAACTACTCGATTCACG	*Xho*I

Restriction enzyme cleavage sites are shown in small letters, and complementary sequences of the primer pairs used for overlap-extension PCR are shown in italics.

### Construction of *C. glutamicum pknG* deletion mutants

*C. glutamicum* ATCC 13032 was transformed with pTM44-ΔpknG using a Gene Pulser Xcell electroporator (Bio-Rad, Richmond, CA, USA) (2.5 kV, 25 μF electric pulse in a 0.1-cm cuvette) followed by heat shock at 46°C for 6 min. The cells were then incubated in 1 mL of BHI medium at 30°C for 1.5 hours. After cultivation for 2 days at 30°C on BHI agar plates containing 25 μg mL^−1^ kanamycin, the transformants were selected for a strain with a single crossover of the Δ*pknG* genotype, which was then cultivated in 5 mL of BHI liquid medium at 30°C overnight and diluted 1:10,000 with MM medium (see below) containing 10% sucrose. The culture was plated on MM agar medium containing 10% sucrose and incubated for 2 days at 30°C. MM medium contains 1 g Yeast Extract, 10 g (NH_4_)_2_SO_4_, 1 g KH_2_PO_4_, 3 g urea, 0.4 g MgSO_4_∙7H_2_O, 2 mg FeSO_4_∙7H_2_O, 2 mg MnSO_4_∙5H_2_O, 0.05 g NaCl, 0.2 mg thiamine, and 0.05 mg biotin per liter. The occurrence of a double-crossover Δ*pknG* mutant was confirmed by its inability to grow after 1 day at 30°C on MM agar containing 25 μg mL^−1^ kanamycin. The sizes of Δ*pknG* (1.60-kbp) in the *C. glutamicum* genome was confirmed using directed PCR with the primer pairs used for the construction and KOD FX (Toyobo). The selected double-crossover strain was designated *C. glutamicum* Δ*pknG*.

### Construction of *C. glutamicum* mutants that express GAD

The construction of the GAD-expression plasmid pCH-gadB, *C. glutamicum* GAD (strain ATCC 13032 harboring pCH-gadB), and *C. glutamicum* W (strain ATCC13032 harboring pCH) was reported (Takahashi et al. [2012]). Plasmid pCH is an *E. coli-C. glutamicum* shuttle vector that drives gene expression with a highly active constitutive promoter (Tateno et al. [2007]). The pCH-gadB construct was introduced into *C. glutamicum* Δ*pknG*. Transformants were selected by growth on BHI agar containing 25 μg mL^−1^ kanamycin. The presence of *gadB* was confirmed using directed PCR with the primer pair SacI-gadB-F and XhoI-gadBF-R. The resulting strain, *C. glutamicum* Δ*pknG* (pCH-gadB) was designated *C. glutamicum* GADΔ*pknG*.

### Western blotting analysis

*C. glutamicum* strains W, GAD, and GADΔ*pknG* were cultured in test tubes at 30°C for 24 hours in 5 mL BHI medium containing 25 μg mL^−1^ kanamycin. Each culture (0.2 mL) was transferred to 20 mL BHI medium containing 25 μg mL^−1^ kanamycin in a 200 mL shaker flask. After fermentation for 24 hours, the cells from a 1 mL culture were centrifuged at 8,000 × *g* for 5 min, washed once in 50 mM Tris–HCl (pH 6.8) buffer, suspended in 1 mL of this buffer, and then 0.7 g of 0.1-mm diameter glass beads YGB01 (Yasui Kikai, Japan) was added to the tube. The cells were disrupted using a Shake Master Neo (Bio Medical Science) by shaking the tube three times at 1,500 rpm for 1 min at 1-min intervals. After centrifugation at 9,000 × *g* for 5 min, the supernatants were subjected to sodium dodecyl sulfate polyacrylamide gel electrophoresis analysis. The separated proteins were electroblotted onto a polyvinylidene fluoride membrane (Millipore, Boston, MA, USA) and then reacted sequentially with a mouse anti-FLAG M2 monoclonal antibody (Sigma, St. Louis, MO, USA) and a goat anti-mouse IgG alkaline phosphate conjugate (Promega) secondary antibody. The membrane was stained with 4-nitro-blue tetrazolium chloride (Promega) and 5-bromo-4-chloro-3-indolyl phosphate (Promega) according to the manufacturer’s instructions.

### Culture conditions for GABA fermentation from glucose

To produce GABA, *C. glutamicum* GAD and the mutant strains were cultured in a test tube at 30°C for 22 hours in 5 mL BHI medium containing 25 μg mL^−1^ kanamycin. This culture (0.2 ml) was transferred to a 200 mL shaker flask containing 20 mL GABA Production 2 (GP2) medium with 25 μg mL^−1^ of kanamycin. GP2 medium contains 50 g glucose, 50 g (NH_4_)_2_SO_4_, 1 g K_2_HPO_4_, 3 g urea, 0.4 g MgSO_4_∙7H_2_O, 50 g soypeptone, 0.01 g FeSO_4_∙7H_2_O, 0.01 g MnSO_4_∙5H_2_O, 200 μg thiamine, 0.5 mg biotin, and 0.265 g pyridoxal 5′-phosphate (PLP) L^−1^. Stock solutions of thiamine, biotin, and PLP were filtered through a 0.22 μm membrane and added to the medium before adding cells. The initial pH of the GP2 medium was 6.30. The pH of the GP2 medium was not adjusted during the fermentation. The fermentation was performed in a BR-13FR BioShaker (Taitec, Japan) at 30°C at 120 rpm.

**Figure 1:**
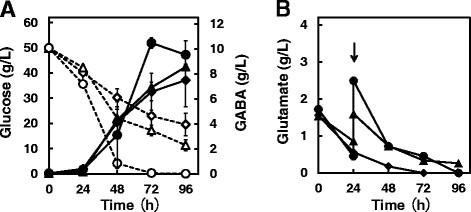
**Effect of glutamate on GABA production by*C. glutamicum*GAD.** To examine the effect of adding L-glutamate during GABA fermentation, *C. glutamicum* GAD was cultured in 20 mL GP2 medium containing 25 μg mL^−1^ kanamycin in a shaker flask at 30°C for 96 hours. At 24 hours, 1.0 g L^−1^ (triangle) or 2.0 g L^−1^ (circle) of L-glutamate was added to the culture. GP2 medium without glutamate (diamond) served as a control. **A**: Extracellular GABA (solid line) and glucose (dotted line) levels in each flask were monitored for 96 hours. **B**: Extracellular glutamate production by *C. glutamicum* GAD in each flask was monitored throughout the fermentation. Data are expressed as the mean and standard error from three independent experiments.

**Figure 2:**
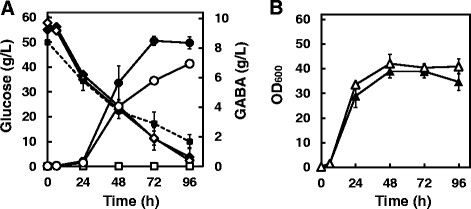
**Time course of extracellular GABA production by*C. glutamicum*strains GAD ∆*pknG*,*C. glutamicum*GAD, and*C. glutamicum*W.** The three strains were cultured separately in 20 mL of GP2 medium containing 50 g L^−1^ glucose and 25 μg mL^−1^ kanamycin using a 200-mL shaker flask. Fermentation was performed at 30°C for 96 hours at 120 rpm. **A**: Extracellular GABA concentrations (circles) and glucose consumption (diamonds) of *C. glutamicum* strains GAD ∆*pknG* (closed symbols) and GAD (open symbols) were monitored. GABA (squares) and glucose consumption (dotted line) of strain W were monitored as controls. **B**: The OD_600_ values of *C. glutamicum* strains GAD ∆*pknG* (closed triangles) and GAD (open triangles) were monitored throughout the fermentation. Data are expressed as the mean and standard error from three independent experiments.

To determine the effect of adding glutamate (Figure 1), *C. glutamicum* GAD was cultured for 96 hours, and 1 g L^−1^ or 2 g L^−1^ of glutamate was added to the culture at 24 hours. To determine the effect of the *pknG* deletion on GABA production (Figure 2), *C. glutamicum* GAD and *C. glutamicum* GAD Δ*pknG* were cultivated for 96 hours. *C. glutamicum* W served as a control.

**Figure 3:**
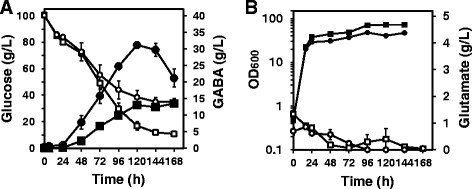
**Time course of extracellular GABA production by*C. glutamicum*GAD, and GAD∆*pknG*from 100 g L**^**−1**^**glucose.***C. glutamicum* strains GAD, and GAD∆*pknG* were cultured separately in 20 mL of GP2 medium containing 100 g L^−1^ glucose and 25 μg mL^−1^ kanamycin in 200-mL baffled flasks. Fermentation was performed at 30°C for 168 hours at 120 rpm. **A**: Extracellular GABA concentrations (closed symbols) and glucose concentrations (open symbols) of *C. glutamicum* strains GAD (squares) and GAD∆*pknG* (circles ) were monitored. **B**: Extracellular glutamate concentrations (open symbols) and the OD_600_ (closed symbols) of the *C. glutamicum* strains GAD (squares), and GAD∆*pknG* (circles) were monitored throughout the fermentation. Data are expressed as the mean and standard error from three independent experiments.

To determine the yield of GABA, fermentation was performed using strains GAD and GADΔ*pknG*. The strains were cultivated in BHI medium at 30°C for 24 hours, and 5% (w/v) of the starter-culture solution was transferred to a 200 mL baffled flask containing 20 mL GP2 medium with 100 g L^−1^ glucose and 25 μg mL^−1^ kanamycin, and agitated at 120 rpm for 168 hours (Figure 3).

Throughout cultivation, 1 ml of the culture was collected from each flask every 24 hours, centrifuged at 8,000 × *g* for 5 min at 4°C, and filtered through a 0.45 μm DISMIC Mixed Cellulose Ester (Advantec, Tokyo, Japan). The concentrations of GABA, glutamate, and glucose in culture supernatants were analyzed as described below. The optical density at 600 nm (OD_600_) was monitored simultaneously.

### Analysis of cell growth, production of GABA and L-glutamate, and consumption of glucose

The growth of the *C. glutamicum* strains was monitored at OD_600_ using a UVmini-1240 UV–vis spectrophotometer (Shimadzu, Kyoto, Japan). GABA and L-glutamate concentrations in the supernatant were analyzed using a Shim-pack Amino-Li column (0.5 μm, 100 mm × 6.0 mm I.D, Shimadzu) and a Prominence Amino Acid Analyzer System (Shimadzu) after derivatization with *ortho*-phthalaldehyde. The mobile phase (lithium citrate-borate gradient ranging from pH 2.68–10.00) was delivered at 0.6 mL min^−1^ at 39°C. Amino acid mixtures Type AN-II and Type B (Wako Chemicals, Japan), 0.1 mM GABA (Nacalai Tesque), and 0.1 mM L-glutamate in sodium citrate buffer (pH 2.2) served as standards. Glucose concentrations were determined using a Prominence HPLC System (Shimadzu) equipped with an Shim-Pac SPR-Pb column (0.5 μm, 250 mm × 4.0 mm I.D., Shimadzu). Water served as the mobile phase and was delivered at a flow rate of 0.6 mL min^−1^ at 80°C. The elution profile was monitored using a refractometer.

## Results

### Effect of exogenous L-glutamate on GABA fermentation by *C. glutamicum* GAD

To examine the effect of glutamate as a precursor for GABA synthesis, L-glutamate was added to a culture of *C. glutamicum* GAD grown on GP2 medium with glucose as the primary carbon source, soy peptone as the nitrogen source, and the GAD cofactor PLP (Figure 1). The culture supernatant was periodically assayed for GABA, glucose, and glutamate (Figure 1). L-Glutamate was added to the culture of *C. glutamicum* GAD after 24 hours, and a parallel control culture lacked added L-glutamate (Figure 1B). The concentrations of GABA after 96 h of fermentation were 9.52 ± 1.14, 8.55 ± 0.2, and 7.49 ± 2.14 g L^−1^ in media containing either 2 g L^−1^, 1 g L^−1^ L-glutamate, or no glutamate, respectively. The maximum concentration of GABA produced by strain GAD in the presence of 2 g L^−1^ L-glutamate was 10.47 ± 0.41 g L^−1^ after 72 h (Figure 1A). The glutamate concentration in the medium of each culture decreased after 24 hours (Figure 1B), and the addition of glutamate was effective for prolonging GABA production.

### Expression of GAD by *pknG* deletion mutants

*C. glutamicum* Δ*pknG* was constructed as described in the Materials and methods section. The GAD-expression plasmid pCH-gadB was introduced into *C. glutamicum* Δ*pknG*, and the resultant recombinant strain harboring pCH-gadB was named *C. glutamicum* GADΔ*pknG. C. glutamicum* strains GAD and W served as controls. The intracellular expression levels of GAD in the engineered *C. glutamicum* strains were monitored by western blotting analysis using an antibody raised against the FLAG-tagged sequence that was incorporated into the cloned genes. The 53 kDa GadB band was detected in the cytoplasmic fractions prepared from *C. glutamicum* strains GAD, GADΔ*pknG,* but not strain W (data not shown).

### Influence of the *pknG* deletion on GABA synthesis

We next examined the effect of the *pknG* deletion on GABA production from glucose, using *C. glutamicum* strains GAD, GADΔ*pknG*, and W*.* As glucose was consumed, GABA formation was detected in the media from cultures of each during stationary phase (Figure 2A). The GABA concentration in the supernatants of cultures of strain GADΔ*pknG* reached 8.48 ± 0.30 g L^−1^ in 72 hours while strain GAD produced 5.79 ± 0.20 g L^−1^ of GABA (Figure 2A). The yield of GABA produced by strain GADΔ*pknG* was 1.46-fold higher compared with that of strain GAD, suggesting that the *pknG* deletion reduced ODHC activity, causing an increase in GABA synthesis. Under these fermentation conditions, strains GADΔ*pknG* and GAD consumed 44.92 g L^−1^ and 46.39 g L^−1^ of glucose within 72 hours, respectively (Figure 2A). The yields of GABA from glucose by strains GADΔ*pknG* and GAD reached 0.337 mol mol^−1^ and 0.233 mol mol^−1^, respectively, in 72 hours. The growth rates of strain GADΔ*pknG* were lower than that of strain GAD (Figure 2B). GABA formation was not observed in the culture medium of strain W (Figure 2A).

### GABA fermentation by GAD and GAD∆*pknG*

To evaluate GABA production, strains GAD, and GAD∆*pknG* were separately cultivated in GP2 medium containing 100 g L^−1^ of glucose using baffled flasks (Figure 3). As glucose in the GP2 medium consistently decreased from the beginning of the fermentation, the concentration of extracellular GABA produced by *C. glutamicum* GAD∆*pknG* simultaneously increased, reaching a maximum level of 31.16 ± 0.41 g L^−1^ after 120 hours (Figure 3A). The rate of GABA production by *C. glutamicum* GAD∆*pknG* reached 0.259 (g L^−1^ h^−1^). As 60.90 ± 4.89 g L^−1^ of glucose was consumed by GAD∆*pknG* in 120 hours, the molar yield of GABA from glucose reached 0.893 mol mol^−1^ (Table 3). At the same time, strain GAD produced 13.06 ± 0.45 g L^−1^ of GABA in 120 hours, consuming 83.62 ± 2.92 g L^−1^ of glucose (Figure 3A, Table 3), The glucose consumption rate of strain GAD∆*pknG* was lower than that of strain GAD (Figure 3A). The molar yield of GABA from glucose by strain GAD was 0.272 mol mol^−1^ in 120 hours. Therefore, the yield of GABA produced by *C. glutamicum* GAD∆*pknG* increased 2.29-fold compared with that of *C. glutamicum* GAD (Table 3). The growth rate of strain GADΔ*pknG* was lower than that of strain GAD. Extracellular L-glutamate was not produced by either strain GAD or GAD∆*pknG* using these fermentation conditions (Figure 3B)*.*

**Table 3:** **Growth (OD600), GABA formation (gL**^**−1**^**, gL**^**−1**^ **h**^**−1**^**) and yield (mol GABA mol glucose**^**−1**^**) of*C. glutamicum*strains producing GABA for 120 hours**

***C. glutamicum*** **strain**	**GAD**	**GADΔ** ***pknG***
OD600	71.86 ± 0.59	40.56 ± 1.05
Glucose consumed (gL^−1^)	83.62 ± 2.92	60.90 ± 4.89
GABA (gL^−1^)	13.06 ± 0.45	31.16 ± 0.41
Relative difference	1	2.29
GABA (gL^−1^ h^−1^)	0.108	0.259
Yield (mol GABA mol glucose^−1^)	0.272	0.893

## Discussion

In the present study, we established a robust system for producing GABA by deleting *pknG* from a strain of *C. glutamicum* that overexpresses GAD. In our previous study, GAD was introduced into wild-type *C. glutamicum*, because it overproduces the GABA precursor L-glutamate from sugar, whereas GABA is produced by *C. glutamicum* GAD directly from glucose. In our optimized conditions for GABA fermentation, GP2 medium contains biotin to support growth. The production of GABA suggests that intracellular glutamate is converted to GABA by strain GAD (Takahashi et al. [2012]). Wild-type *C. glutamicum* does not produce glutamate under ordinary culture conditions unless glutamate secretion is induced by culturing the biotin-auxotrophic wild-type strain in biotin-limiting conditions (Shiio et al. [1962]). Moreover, when L-glutamate was added to cultures of *C. glutamicum* GAD to determine its effect on GABA fermentation, an increase in the levels of GABA in the medium was observed when 2 g L^−1^ of L-glutamate was added (Figure 1A). We reasoned that because glutamate is a precursor in the synthesis of GABA, its increased availability would enhance the yield of GABA. Based on this rationale, we were able to successfully generate a *C. glutamicum* mutant that produced relatively high levels of GABA.

We focused on *pknG*, because its product (PknG) regulates the activity of ODHC. PknG activates ODHC by phosphorylating its subunit OdhI, which is a subunit of ODHC. ODHC acts at a branch point of the TCA cycle where it catalyzes the conversion of 2-oxoglutarate to succinyl-CoA. Unphosphorylated OdhI inhibits the ODHC activity of *C. glutamicum* (Niebisch et al. [2006]). A *pknG*-deficient mutant of *C. glutamicum* produces 4.3-fold higher amounts of glutamate compared with wild-type under biotin-limiting conditions (Schultz et al. [2007]). The *C. glutamicum* 2262 *pknG* mutant also produces glutamate at a 40% higher specific rate compared with wild-type (Boulahya et al. [2010]). Therefore, a *pknG*-deficient strain was constructed to reduce the metabolic flux to the TCA cycle.

We show here that the yield of GABA in cultures of strain GAD∆*pknG* was 2.29-fold higher in 120 hours compared with that of strain GAD (Figure 3, Table 3), suggesting that the *pknG* deletion influenced ODHC activity by causing an increase in the intracellular glutamate level that enhanced GABA production. We assumed that the ODHC activity of strain GAD∆*pknG* was reduced, because OdhI was not phosphorylated and could not activate the ODHC complex, which caused an increase in carbon flux into the glutamate pathway compared with that of strain GAD. The glucose consumption rate and growth rate of GAD∆*pknG* was lower than that of GAD (Figure 3, Table 3)*,* suggesting that the flux to TCA cycle was decreased. We plan to analyze carbon flux of these strains in the future. Moreover, in the late stage of fermentation, reduction of GABA production was observed in cultures of strain GAD∆*pknG* (Figure 3A). Because reduced levels of the product were also observed in cultures of strain GAD (Takahashi et al. [2012]), we are now attempting to disrupt the genes for GABA assimilation.

In our GABA production system using *C. glutamicum* GADΔ*pknG*, high concentrations of GABA were produced from glucose in GP2 medium without the addition of glutamate. The yield of GABA from glucose produced by strain GADΔ*pknG* reached 0.893 mol mol^−1^, and the highest yield was produced in 120 hours (Table 3). Using *C. glutamicum* GADΔ*pknG*, we expect that fewer fermentation by-products will be produced and that the recovery of GABA will be simpler than using methods for its isolation from cultures of wild-type lactic acid bacteria.

GABA is primarily produced using cultures of lactic acid bacteria containing glutamate or monosodium glutamate (MSG) (Li and Cao [2010]). For example, *Streptococcus salivarius* subsp. *thermophilus* Y2, a cheese starter strain, produces 7.98 g L^−1^ of GABA after 84 hours of fermentation with a continuous supply of 15 g L^−1^ MSG, corresponding to a rate of 0.095 g L^−1^ hour^−1^ (Lu et al. [2008]). *L. paracasei* NFRI 7415, which was isolated from fermented fish, produces 31.11 g L^−1^ (302 mM) GABA in 168 hours, corresponding with a production rate of 0.185 g L^−1^ hour^−1^. Although the production rate by strain NFRI 7415 was relatively high, 500 mM (73.5 g L^−1^) glutamate was added to the culture medium (Shima et al. [2005]). *L. brevis* NCL912, which was isolated from Paocai, produces 345.83 mM (35.6 g L^−1^) GABA in a medium containing 500 mM glutamate (Cao et al. [2010]). Microbial production systems that require supplementation with amino acids are not cost-effective for applications such as synthesizing chemicals. In contrast, in our GABA production system using *C. glutamicum* GADΔ*pknG,* high concentrations of GABA were produced from glucose in one step (31.16 g L^−1^ from glucose at 0.259 g L^−1^ h^−1^ and therefore will provide a new platform for synthesizing chemicals.

**Figure 4:**
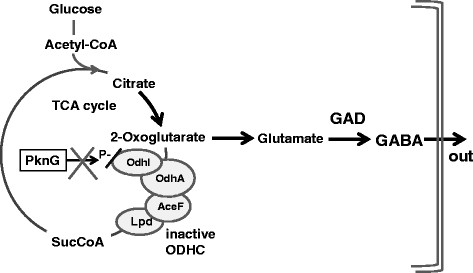
**Model of direct GABA production by** ***C. glutamicum*** **GAD∆** ***pknG*.**

Recently, two GADs from *L. brevis* were expressed in *C. glutamicum* ATCC 13032/pDXW-8-gadRBC2, which produced 27.13 ± 0.54 g L^−1^ of GABA from glucose in 120 hours using flask fermentation with six urea supplements to the medium during the fermentation (Shi et al. [2013]). With urea supplementation, increased amounts of glutamate were produced in the culture supernatant at the same time; however, it may be difficult to separate the product from the medium. In our system, 31.16 g L^−1^ of GABA was directly produced from glucose without addition of a nitrogen or carbon source during the fermentation. Notably, because our GP2 medium contains biotin to support growth, glutamate is not secreted (Figures 3B and 4). A one-step production system has long been a goal for producing precursors for synthesizing bulk chemicals, and we show here that this was possible for robust production of GABA using GADΔ*pknG.*

Our future work involves the development of a process to produce GABA from abundantly available starch or cellulose. We developed a system for coexpressing amylase and lysine decarboxylase in *C. glutamicum* to produce cadaverine from soluble starch (Tateno et al. [2009]). Further, our *C. glutamicum* endoglucanase secretion systems for producing glutamate from beta glucan (Tsuchidate et al. [2011]) can be applied to the production of GABA. The production of GABA using strains based on *C. glutamicum* GADΔ*pknG* would allow the synthesis of 100% biomass-derived nylon PA4. Notably, *C. glutamicum* is generally recognized as safe (GRAS) according the United States Food and Drug Administration. Therefore, the system for GABA fermentation developed in the present study can be applied to the production of GABA as a component of foods and pharmaceuticals.

## Competing interests

The authors declare that they have no competing interests.
